# Synthesis and Structural and Optical Behavior of Dehydrohelicene-Containing Polycyclic Compounds

**DOI:** 10.3390/molecules29020296

**Published:** 2024-01-05

**Authors:** Md. Imrul Khalid, Mohamed S. H. Salem, Shinobu Takizawa

**Affiliations:** 1 SANKEN, Osaka University, Mihogaoka, Ibaraki-shi 567-0047, Osaka, Japan; imrul43@sanken.osaka-u.ac.jp (M.I.K.); mohamedsalem43@sanken.osaka-u.ac.jp (M.S.H.S.); 2 Organic and Carbon Nanomaterials Unit, Okinawa Institute of Science and Technology Graduate University, 1919-1 Tancha, Onna-son, Kunigami-gun, Okinawa 904-0495, Japan; 3 Pharmaceutical Organic Chemistry Department, Faculty of Pharmacy, Suez Canal University, Ismailia 41522, Egypt

**Keywords:** dehydrohelicene, quasicirculene, helicenes, circulenes, nanographene, material-based applications, Scholl reaction, photophysical, chiroptical, synthesis

## Abstract

Dehydrohelicene-based molecules stand out as highly promising scaffolds and captivating chiroptical materials, characterized by their unique chirality. Their quasi-helical π-conjugated molecular architecture, featuring successively *ortho*-annulated aromatic rings, endows them with remarkable thermal stability and optical properties. Over the past decade, diverse approaches have emerged for synthesizing these scaffolds, reinvigorating this field, with anticipated increased attention in the coming years. This review provides a comprehensive overview of the historical evolution of dehydrohelicene chemistry since the pioneering work of Zander and Franke in 1969 and highlights recent advancements in the synthesis of various molecules incorporating dehydrohelicene motifs. We elucidate the intriguing structural features and optical merits of these molecules, occasionally drawing comparisons with their helicene or circulene analogs to underscore the significance of the bond between the helical termini.

## 1. Introduction

**Figure 1:**
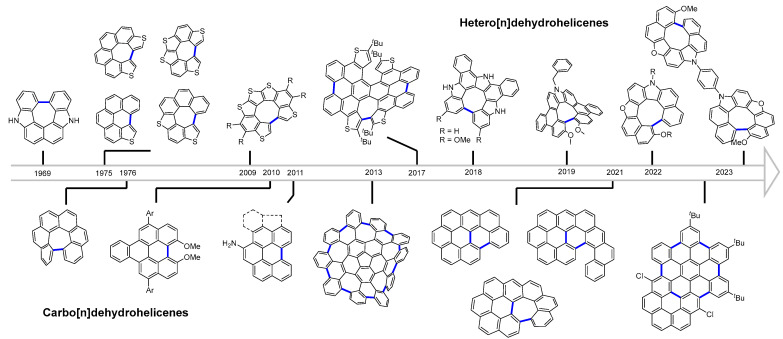
Comprehensive overview of the pivotal accomplishments in the chemistry of carbo[*n*]dehydrohelicenes and hetero[*n*]dehydrohelicenes over last 50 years: (**upper portion**) hetero[*n*]helicenes, distinguished by their incorporation of sulfur, nitrogen, or oxygen atoms into the helical framework, adding an extra layer of complexity and functionality; (**lower portion**) carbo[*n*]dehydrohelicenes, characterized by a dehydrohelicene scaffold composed solely of carbon and hydrogen atoms. Blue color indicates the sigma bonds connecting the two helical termini of dehydrohelicenes.

The exploration of stereochemistry has been a subject of enduring interest within the chemical community since Pasteur’s landmark discovery of molecular chirality in the 19th century [1], followed by van ‘t Hoff and Le Bel’s influential introduction of tetrahedral carbon [2,3]. Within the realm of molecular chirality, various types can be distinguished, including the well-known central, axial, helical, and planar chirality [4]. Twisting some one-dimensional nanocarbons out of planarity affects their optical and electronic properties and also induces chirality as shown in helically locked tethered twistacenes (twisted acenes) [5,6,7]. Certain compounds pose a challenge when attempting to classify them as purely axial or helical, as they exhibit characteristics of both. These molecules possess a rotational axis that contributes to their chirality, while simultaneously displaying a helical or spiral arrangement [8]. The most famous example of such scaffolds is the dehydrohelicenes, also described as quasicirculenes, which are *ortho*-fused polycyclic aromatic compounds in which the two helical termini are connected by a sigma bond [9,10,11]. They are derived from helicenes, which are compounds with a series of fused benzene or heterocyclic rings arranged in a helical or screw-like structure [12,13,14,15,16]. Dehydrohelicenes specifically contain double bonds in their helical structure, which introduces additional conjugation and alters their electronic properties compared to their fully saturated counterparts. Their unique scaffolds and chirality endowed them with various desirable photophysical, chiroptical, and electronic features that can be implemented in different material-based applications [17,18]. Although the first example of dehydrohelicenes was reported 50 years ago by Zander and Franke, attention to their impact was not grabbed until recently [19]. This may be attributed to the fact that most dehydrohelicenes have been considered for a long time as side products in our quest for circulenes [20]. With the recent remarkable developments in the fields of renewable energy and materials applications of small organic molecules (SOMs) during the last two decades [21,22,23,24], dehydrohelicenes’ characteristics were revisited, and their potential was re-evaluated for further innovative applications. Additionally, the advancements in measuring tools and chiroptical methods, such as Raman optical activity (ROA) [25], vibrational circular dichroism (VCD) [26,27,28], and circularly polarized luminescence (CPL) facilitated extensive investigations of the electronic and geometric merits of dehydrohelicenes, enabling a deeper understanding of their value [22,23,29,30,31]. Given their higher chiroptical responses and chiral stabilities, in many reported examples, compared to their helicene analogs, dehydrohelicenes received great attention in the last decade and various synthetic approaches were developed to access these scaffolds [10]. Dehydrohelicenes can be classified based on the incorporation of heteroatoms in their main skeleton into carbo-dehydrohelicenes and hetero-dehydrohelicenes (Figure 1). The first has a purely carbon-based ring structure, while the latter have heteroatoms incorporated into their ring, adding an extra layer of complexity and functionality [32,33,34].

## 2. Carbo[*n*]dehydrohelicenes

Carbo[*n*]dehydrohelicenes represent a distinctive class of polycyclic aromatic hydrocarbons (PAHs) characterized by a cyclic scaffold featuring one or more double bonds arranged in a strained configuration. Notably, the structural composition of carbo[*n*]dehydrohelicenes is entirely carbon-based, devoid of any heteroatoms, wherein the constituent atoms are exclusively carbon [20]. This particular subset of dehydrohelicenes has saved considerable interest within the realm of organic chemistry, primarily owing to its exceptional molecular architecture [35]. In the burgeoning realm of nanographenes, their potential applications across diverse fields such as electronics, optoelectronics, and materials science have sparked a transformative revolution [36,37]. The controlled synthesis of nanographenes presents a dual challenge and opportunity, allowing precise adjustment of properties based on size, shape, and functionalization [38,39,40,41]. Noteworthy, attention has recently been redirected to carbo[*n*]dehydrohelicenes, recognized as promising candidates due to their twisted structures, extensive conjugation, and inherent chirality in some cases [35,42]. The helical arrangement of dehydrohelicenes, driven by steric constraints and enhanced by optical activity, offers unique possibilities in asymmetric synthesis and as components for chiral sensors. Additionally, the absence of heteroatoms in their scaffold imparts unique chemical and physical properties, rendering this class of compounds particularly intriguing for exploration in the domains of materials science and bottom-up synthetic chemistry [43,44,45]. In the following sections, we will elucidate the seminal progressions in the synthesis and applications of carbo dehydrohelicenes, stratified according to the number of constituent rings within each dehydrohelicene scaffold.

### 2.1. Carbo[5]dehydrohelicenes

**Scheme 1:**
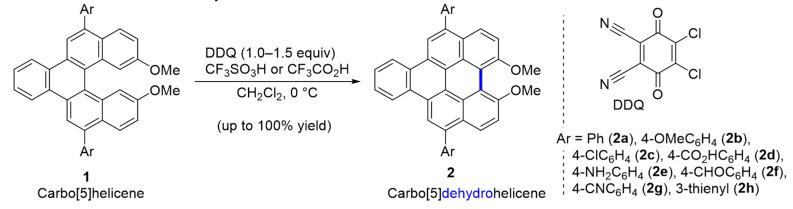
Synthesis of carbo[5]dehydrohelicene derivatives via Scholl reaction.

In a significant scientific investigation conducted by Chen and colleagues in 2010, the synthesis of carbo[5]dehydrohelicene **2** from the corresponding carbo[5]helicene derivatives **1** was reported [46]. The authors determined that the oxidative cyclodehydrogenation reactions of the helicenes **1** could be effectively achieved by employing the conditions established for Scholl reactions [47]. By utilizing either trifluoroacetic acid or trifluoromethanesulfonic acid with 2,3-dichloro-5,6-dicyano-1,4-benzoquinone (DDQ), carbo[5]helicene derivatives **1** was oxidized to form carbo[5]dehydrohelicenes **2** in excellent yields (Scheme 1).

**Scheme 2:**
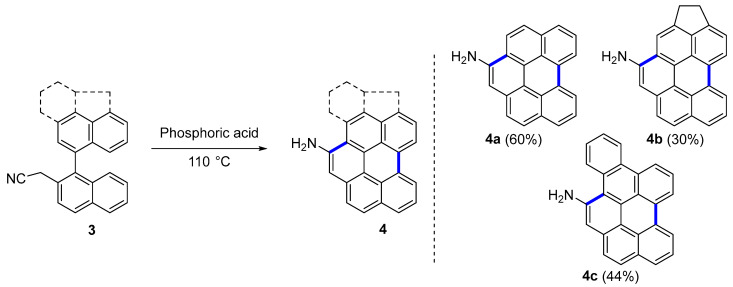
Direct access to the unique architectures of amino benzo[*ghi*]perylene.

In 2011, Gaucher, Giner Planas, and coworkers reported novel molecular scaffolds of benzo[*ghi*]perylene derivatives **4** via a one-pot electrophilic aromatic substitution—Scholl reaction sequence. Under acidic conditions, the cyanomethylene pendant arm was installed on the binaphthyl moiety of **3** to promote the generation of the corresponding electrophile and ultimately give planar amino benzo[*ghi*]perylene units classified as carbo[5]dehydrohelicenes **4** in moderate to good yields (Scheme 2). Unlike their expectations, the polyphosphoric acid (PPA) was not able to afford the corresponding carbo[5]dehydrohelicenes **4** even at elevated temperatures 110 °C. The synergy of carborane and amino benzo[*ghi*]perylene moieties yielded distinctive 3D-planar molecular architectures. Photophysical analysis showcased that, while the absorption profile was primarily governed by the perylene-rigid fragment, both the carborane and organic subunits intricately influenced the emission characteristics of these novel structures [48].

**Scheme 3:**
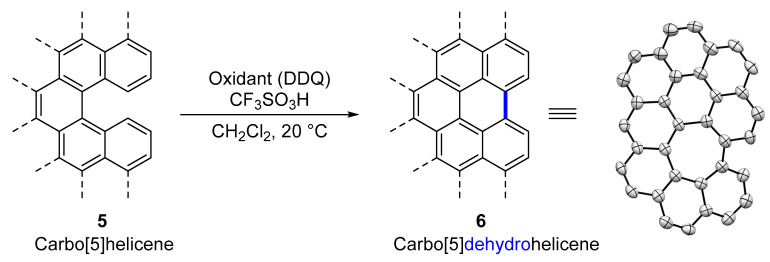
Oxidative cyclo-rearrangement of carbo[*n*]helicenes into their corresponding carbo[5]dehydrohelicenes.

More than 10 years later, Qiu et al. in 2021 developed a fascinating strategy to broaden the diversity of many carbo[*n*]helicenes **5** and give direct access to their corresponding carbo[*n*]dehydrohelicenes **6** through a unique oxidative cyclo-rearrangement reaction (Scheme 3). This Scholl cyclization process leads to the fabrication of a series of carbo[5]dehydrohelicene **6** driven by the gradual release of the strain of the highly distorted helicene skeletons. The authors employed a wide variety of acids, including CF_3_CO_2_H, CH_3_SO_3_H, and CF_3_SO_3_H, along with DDQ as a crucial component to promote carbo[*n*]helicenes with (*n* = 7–9) to be firstly oxidized, cyclized, and subsequently rearranged into nanographenes with an unsymmetrical helicoid shape through sequential 1,2-migrations [49].

**Scheme 4:**
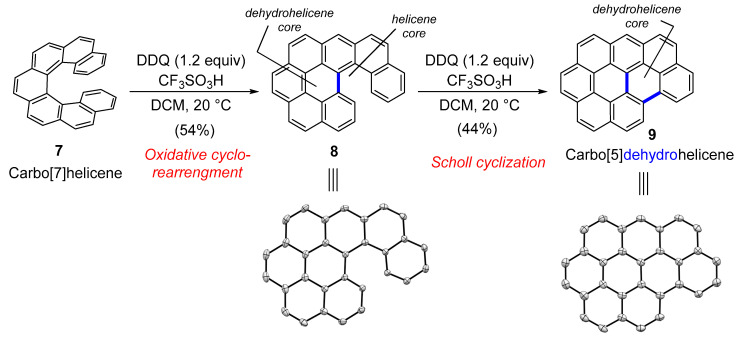
Skeletal reconstruction of carbo[7]helicenes via a two-step oxidation protocol.

Upon oxidation by DDQ in the presence of CF_3_SO_3_H acidic additives, carbo[7]helicene **7** undergoes a cascade of oxidative cyclo-rearrangement followed by a Scholl cyclization reaction to afford the planar analogs of carbo[5]dehydrohelicenes **8** and **9** (Scheme 4) [49].

Most carbo[5]dehydrohelicene derivatives demonstrated commendable solubility in commonly used organic solvents. Compound **2a** exhibited a significant absorption band peaking at 436 nm in cyclohexane, demonstrating a pronounced bathochromic shift of 51 nm compared to benzo[*ghi*]perylene. This shift can be attributed to the extensively conjugated system of **2a** and, in some cases, the electron-donating effect of the methoxy groups in **2b** for example. Moreover, the maximum emission wavelength of **2a** was determined to be 478 nm, aligning with a distinct cyan fluorescence in the fluorescent spectrum [46]. To comprehensively investigate the photophysical properties of the other carbo[5]dehydrohelicene **2b**–**2h**, their UV–VIS absorbance, and photoluminescence (PL) patterns were studied in cyclohexane and exhibited comparable results to those of **2a** (Table 1). Importantly, the non-planarity of some substituted aromatic rings of the carbo[5]dehydrohelicene derivatives **2b** and **2f** resulted in minimal shifts in the emission wavelength. The quantum yields of the carbo[5]dehydrohelicenes varied considerably (Table 1), with electron-withdrawing groups generally yielding lower values, while electron-donating groups yielded higher values.

On the other hand, benzo[*ghi*]perylene derivatives **4** exhibited commendable photophysical properties, as outlined in Table 2, detailing the UV–VIS absorption and emission maxima for compounds **4a**–**4c** in THF. Notably, the incorporation of an electron-donating group, exemplified by –NH_2_ in the structure of benzo[*ghi*]perylene, resulted in a discernible shift and broadening of both absorption and fluorescence spectra relative to pristine benzo[*ghi*]perylene. This observation aligns with the anticipated trends associated with the introduction of such functional groups into the benzo[*ghi*]perylene frameworks [50].

**Scheme 5:**
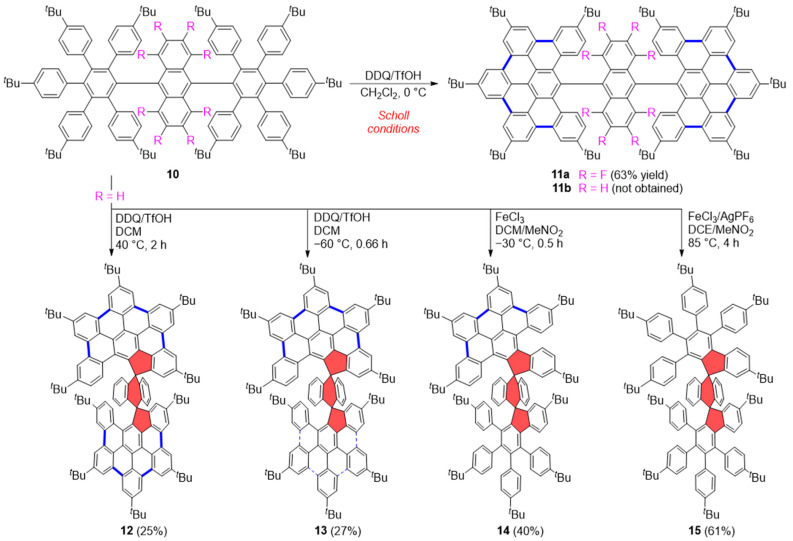
Synthesis of various partially graphitized and fully graphitized spironanographene (dashed lines in compound **13** indicates that one of these bonds is not formed).

In 2023, Fernández and Martín demonstrated an innovative application of the Scholl reaction in the bottom-up synthesis of molecular nanographenes. Their investigation focused on the electronic effects of different starting substrates—specifically, anthracene versus octafluoroanthracene—and its control over the Scholl reaction. The study successfully yielded a series of spirocompounds (**12**–**15**) featuring a dehydrohelicene core with varying degrees of graphitization. The authors initially proposed the hypothetical synthesis of a helically arranged molecular nanographene **11b**, based on a 9,10-substituted anthracene with two dibenzo[fg,ij]phenanthro-[9,10,1,2,3-pqrst]pentaphene (DBPP) units **10b**. However, the optimized Scholl reaction conditions led to the isolation of partially graphitized intermediates (**13**–**15**) and the fully graphitized spironanographene **12** (Scheme 5) [51].

Electrochemical and photophysical investigations of *syn*-**12** unveiled disrupted electronic communication attributed to the emergence of two *sp*^3^ spiranic carbons. The expanded band gap, in comparison with hexa-*peri*-hexabenzocoronene (HBC), manifested in distinctly separated reduction and oxidation potentials, accompanied by electronic bands experiencing a blue shift. Helically arranged nanographene **11a**, displaying acceptor characteristics, exhibited a comparatively lower first reduction potential in the CV, red-shifted bands in absorption and emission spectra, and a dual emission from the octafluoroanthracene fragment and the DBPP moieties [51].

**Scheme 6:**
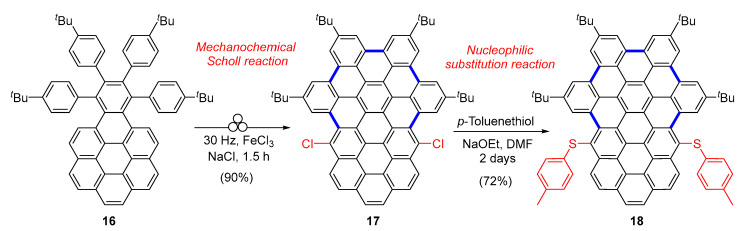
Mechanochemical synthesis of functionalizable curved nanographenes.

In 2023, the Stuparu group reported a groundbreaking synthesis of precisely functionalizable curved nanographenes through graphitization-induced regioselective chlorination in a mechanochemical Scholl reaction [52]. Mechanochemistry, utilizing mechanical energy through grinding or milling, has recently emerged as a powerful synthetic tool, offering a sustainable approach to modifying chemical reactivity and product selectivity [53,54,55,56,57,58,59]. The mechanochemical Scholl reaction, facilitated by iron(III) chloride, demonstrated notable success [57,60,61,62,63], with precursor **16** undergoing regioselective chlorination to produce nanographene **17**. This solvent-free reaction employed a mixer mill with a ZrO_2_ grinder jar and ZrO_2_ balls, and sodium chloride as a milling auxiliary, with milling conducted for 1.5 h at a frequency of 30 Hz (Scheme 6). Characterization via MALDI-TOF mass spectrometry confirmed successful graphitization, revealing bis-chlorination during the process. Intriguingly, the graphitization step activated adjacent positions for chlorination, resulting in chlorinated curved nanographenes. This regioselective chlorination presents an opportunity for atomically precise functionalization of the nanographene scaffold [52].

UV–VIS spectroscopy of curved nanographenes **17** demonstrated moderate-intensity absorption bands at 400–550 nm with a significant 150 nm bathochromic shift compared to corannulene and coronene, indicating extended π-conjugation. The thioether-functionalized nanographene **18** exhibited the most red-shifted absorption band at 510 nm, attributed to the electron-donating nature of sulfur atoms. This observation suggests the potential for tuning the band-gap through post-synthesis functionalization. In fluorescence emission spectroscopy, the nanographenes displayed emissive properties in the blue-green region of the electromagnetic spectrum [52].

### 2.2. Carbo[6]dehydrohelicenes

**Scheme 7:**
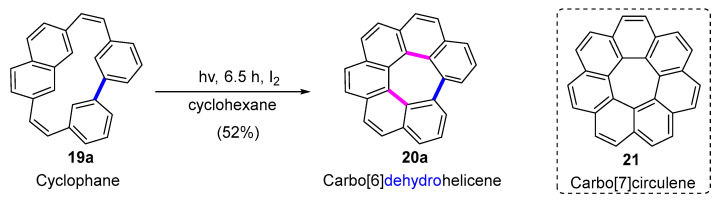
Photochemical synthesis of carbo[6]dehydrohelicene.

Jessup and Reiss got the first carbo[6]dehydrohelicene **20** upon irradiating a solution of the cyclophane diene **19** in the presence of iodine as an oxidant (Scheme 7). It’s noteworthy, their focus was primarily directed towards the synthesis of carbo[7]circulene **21**, and they did not explore the optical or electronic features of this intriguing carbo[6]dehydrohelicene scaffold **20** [20].

**Scheme 8:**
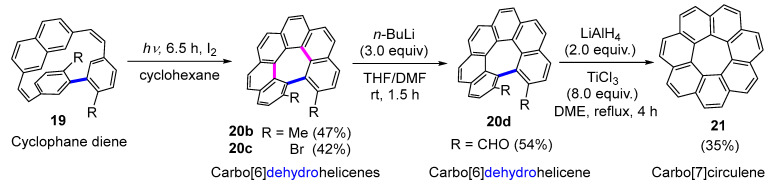
Early synthesis of carbo[7]circulene and some carbo[6]dehydrohelicene derivatives.

Later, Yamamoto and coworkers during their successful preparation of carbo[7]circulene **21**, confirmed the chiral nature of carbo[6]dehydrohelicenes **20**, and introduced another three derivatives of chiral carbo[6]dehydrohelicenes **20b**–**20d** upon irradiation of diene **19** (Scheme 8). However, the moderate racemization barriers of these carbo[6]dehydrohelicenes **20** (calculated barrier = 32.0 kcal mol^−1^) hindered their further study and investigation [42].

**Scheme 9:**
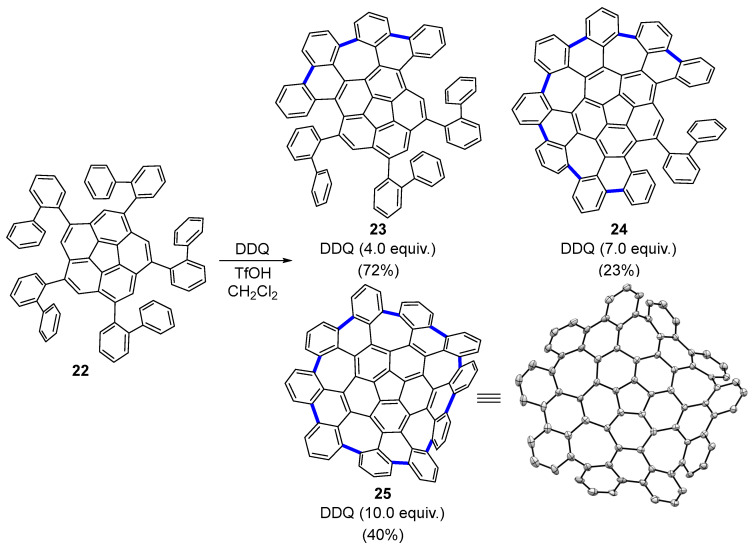
Preparation of a grossly warped nanographene with dehydrohelicene core.

In 2013, Itami and Scott reported an unconventional grossly warped nanographene with a dehydrohelicene core **25**, that showed superior behavior in terms of solubility, optical, and electronic features compared to other planar nanographene. The authors successfully synthesized this compound **25** from its precursor corannulene derivative **22** via Scholl reaction using 10 equivalents of DDQ (Scheme 9). The cyclization process affixes the five polycyclic wing-like substituents to the hydrocarbon core then suture them together to generate ten new C–C bonds and five new seven-membered rings [35].

The distinctive double-concave structure of compound **25** was explained by the dense accumulation of odd-membered-ring defects. The central corannulene moiety adopted a shallow bowl-shaped geometry with a depth of 0.37 Å. Notably, **25** exhibited chirality due to the presence of five helical moieties, each with either *M* or *P* chirality around the seven-membered ring, resulting in enantiomers with (*MPMPM*) and (*PMPMP*) configurations. While 25 exhibited minimal racemization barriers (calculated barrier of only 1.7 kcal mol^−1^), limiting chiroptical investigations, the structural warping significantly enhanced the solubility and exerted prominent effects on both electronic and optical properties [35].

## 3. Hetero[*n*]dehydrohelicenes

Hetero[*n*]dehydrohelicenes, also referred to as quasi-heterocirculenes fall under the category of polycyclic heteroaromatics (PHAs) [17,18]. This distinct class of compounds features a sigma bond connecting the two helical termini of the corresponding hetero[*n*]helicene, resulting in exceptional photophysical, electronic, and chiroptical responses. Some hetero[*n*]dehydrohelicenes have reported a significant enhancement in chiral stability compared to their hetero[*n*]helicene analogs, thereby amplifying their industrial value and broadening their range of applications [10]. These unique characteristics find applications in diverse material-based technologies, including organic light-emitting diodes (OLEDs) and field-effect transistors (FETs). The remarkable chiroptical properties of hetero[*n*]dehydrohelicenes, such as circular dichroism (CD) and circularly polarized luminescence (CPL), open avenues for applications in optical information storage and transfer [64]. The key distinction between hetero[*n*]dehydrohelicenes and their carbo- analogs lies in the incorporation of one or more heteroatoms into the dehydrohelicene scaffolds. This modification results in the modulation of their physical and optical characteristics, consequently altering electronic properties and expanding their utility [65,66,67]. Despite the promising potential in various applications, it is crucial to recognize that the realm of hetero[*n*]dehydrohelicene chemistry represents a relatively recent and burgeoning area of research. Realizing its full potential necessitates comprehensive investigations aimed at unraveling its intricate properties and delineating the scope of its applications across scientific and technological domains [68,69,70].

### 3.1. Hetero[5]dehydrohelicenes and Hetero[6]dehydrohelicenes

**Scheme 10:**
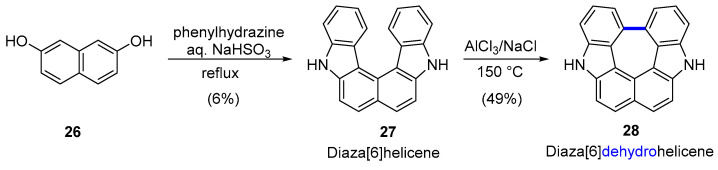
First report of synthesizing dehydrohelicene scaffold using the chloraluminate method.

In 1969, Zander and Franke reported the first example of hetero[*n*]dehydrohelicene structures, representing the initial report among all dehydrohelicene core scaffolds. They employed a Scholl-type reaction to introduce diaza[6]dehydrohelicene **28**, synthesized from the precursor diaza[6]helicene **27** through AlCl_3_-mediated terminal ring closure at 150 °C (Scheme 10) [19]. 

**Scheme 11:**
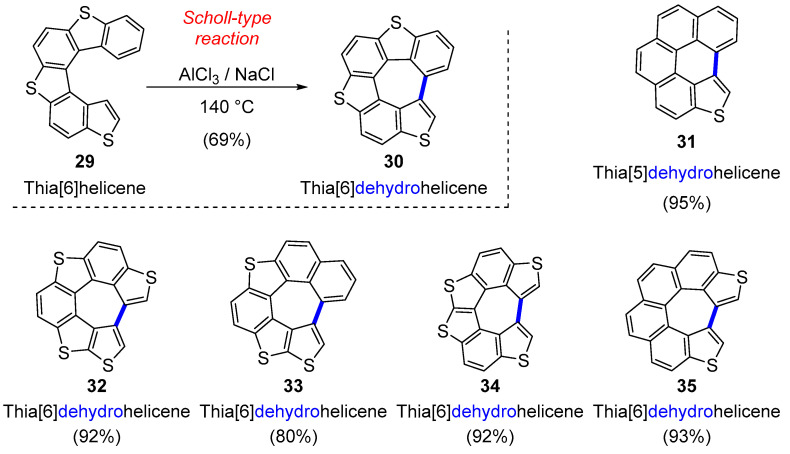
Dehydrogenation of heterohelicenes by a Scholl-type reaction.

In 1975, Wynberg and co-workers used a similar Scholl-type approach to access various thiophene-based dehydro[5]helicenes **31** and dehydro[6]helicenes (**30**, **32**–**35**) in high yields (up to 95%) from the corresponding thia[*n*]helicenes **29** (Scheme 11) [9].

This study stood as one of the pioneering attempts to establish the sigma bond between the two helical termini of diverse hetero[*n*]helicenes, especially multi-thia[*n*]helicenes. Drawing insights from multiple trials and unsuccessful examples documented by the Wynberg group, it was evident that this intramolecular ring closure step was specifically confined to hetero[5]helicenes and hetero[6]helicenes [9,71].

### 3.2. Hetero[7]dehydrohelicenes

**Scheme 12:**
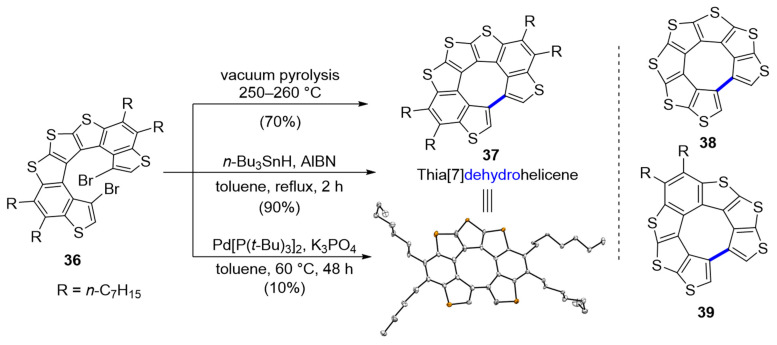
Cyclization of thiophene-based [7]helicene at the two bromine-substituted termini.

In 2009, Rajca described dehydrohelicene derivatives as quasi-circulenes for the first time, after getting three novel thiophene-based dehydro[7]helicene molecules **37**–**39** from their corresponding dibromo[7]helicenes **36** by three different methods; pyrolysis, tin-mediated, or palladium-mediated carbon-carbon bond forming reactions (Scheme 12) [11].

The interest in studying the photophysical properties of hetero[*n*]dehydrohelicenes dates back to 2009, when Rajca and coworkers studied the UV–VIS absorption of their three thiophene-based dehydro[7]helicene derivatives. The studied molecules **37**–**39** revealed good UV–VIS absorption properties characterized by a red-shifting with the increasing number of benzene rings and decreasing number of thiophene rings (Table 3) [11].

**Scheme 13:**
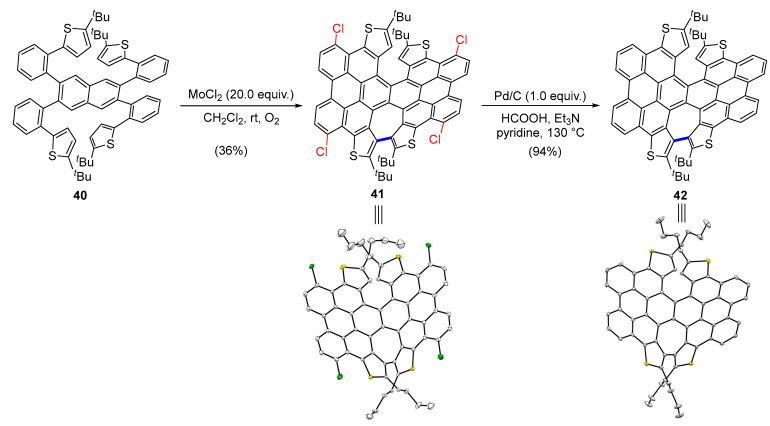
Preparation of saddle-helix hybrid molecule with dehydrohelicene core moiety.

In 2017, Itami and Segawa reported the synthesis of a highly distorted saddle-helix molecule, **42**, with an exceptional racemization barrier (theoretically predicted to be 49.7 kcal·mol^−1^). This represented a significant improvement compared to their previously reported grossly warped nanographene **25** (Scheme 9). The unique structure of **42** enabled the chiral HPLC resolution of both enantiomers for the first time, facilitating subsequent investigations into their chiroptical features. The synthesis involved a stepwise sequence, including a MoCl_2_-mediated oxidative coupling of compound **40** with chlorination to cap reactive sites. Subsequent Pd-catalyzed dechlorination yielded compound **42** (Scheme 13). Electronic states of compounds **41** and **42** were studied using photophysical and electrochemical methods, revealing insights into the effects of chloride atom substitution and heptagonal ring formation [72]. 

The UV–VIS absorption spectra of **41** and **42** displayed two major absorption bands, with vibronic structures in longer-wavelength absorption and intense absorption around 400 nm. The absorption λ_max_ of **41** were observed at 518 nm, 486 nm, 455 nm, and 404 nm. In comparison, these λ_max_ were blue-shifted for **42**, appearing at 502 nm, 472 nm, 442 nm, and 394 nm. Fluorescence spectra exhibited a similar tendency with spectral shift compared to absorption pattern, with maxima at 530 and 566 nm for **41** (Φ_F_ = 0.37) and at 514 and 548 nm for **42** (Φ_F_ = 0.23). The circular dichroism (CD) spectrum displayed negative Cotton effects at 501 nm (Δε = −82 M^−1^·cm^−1^) and 471 nm (Δε = −44 M^−1^·cm^−1^) and positive Cotton effects at 421 nm (Δε = +111 M^−1^·cm^−1^), 398 nm (Δε = +75 M^−1^·cm^−1^), and 326 nm (Δε = +54 M^−1^·cm^−1^) [72].

**Scheme 14:**
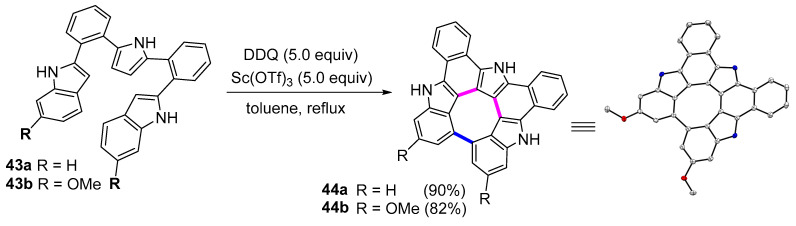
Preparation of triaza[7]dehydrohelicene derivatives with low chiral stability.

In 2018, Tanaka and Osuka introduced new triaza[7]dehydrohelicene molecules **44** that exhibit interesting photophysical properties. These scaffolds were synthesized in high yields via the oxidative fusion reaction of **43** using DDQ-Sc(OTf)_3_ reagents in toluene under reflux conditions (Scheme 14). The UV–VIS absorption spectra of **44a** and **44b** exhibited a red-shifted absorption reaching up to λ_max_ = 470 nm. Additionally, their emission bands were shifted in a bathochromic way at 462 nm and 476 nm for compounds **44a** and **44b**, respectively, with a relatively large Stokes shift (1450 cm^−1^ in case of **44a**) probably due to their non-planar conformations. The very low racemization barriers of these structures (theoretically predicted to be ~5.2–6.0 kcal·mol^−1^) are an obstacle to the further investigation of their chiroptical merits [73].

**Scheme 15:**
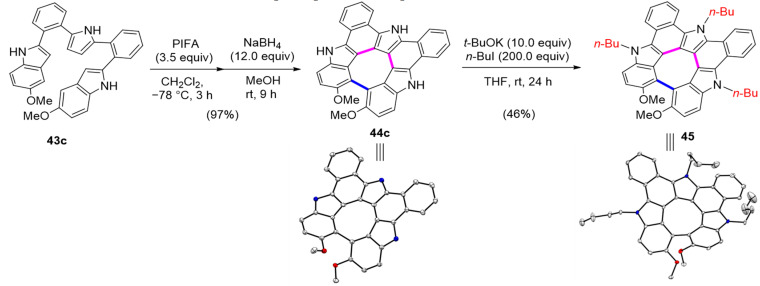
Preparation of modified triaza[7]dehydrohelicene derivatives with high chiral stability.

One year later, the same research group presented a modified iteration of their triaza[7]dehydrohelicene **45**, featuring a significantly high racemization barrier of approximately 40 kcal·mol^−1^. This advancement facilitated chiral resolution through HPLC and allowed an exploration of the compound’s promising chiroptical features (Scheme 15). The CD spectra of **45** exhibited a mirror-image pattern, revealing a negative Cotton effect around 450 nm attributed to the (*M*) twisted form, as confirmed by comparison with the TD-DFT calculated CD spectrum. The UV–VIS absorption spectra of **45** demonstrated red-shifted absorption patterns compared to **44a** [74].

**Scheme 16:**
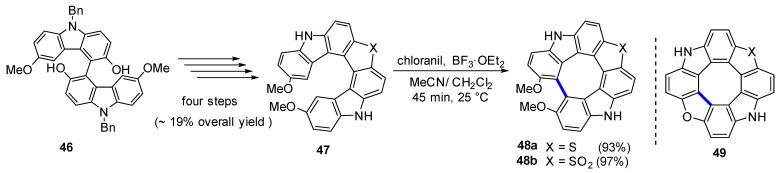
Preparation of unique dehydro[7]helicene derivatives with three different heteroatoms.

In 2020, Pittelkow and coworkers reported two interesting hetero[7]dehydrohelicenes **48a** and **48b** with three different heteroatoms (N, S, and O) incorporated into their scaffold that showed improved optical and electrochemical features compared to the corresponding hetero[7]helicenes **47** and hetero[8]circulenes **49**. These two hetero[7]dehydrohelicenes **48a** and **48b** were synthesized in high yields via an intramolecular Scholl-type reaction upon treatment of the corresponding hetero[7]helicenes **47** with BF_3_·OEt_2_ and chloranil. On the other hand, the helicene precursors **47** required a long synthetic protocol to be afforded from readily available substrates over four steps and affording 19% overall yield (Scheme 16). The photophysical properties, UV–VIS absorption and PL of these series were investigated in THF to reveal that the planarization of the scaffold showed a big impact on the photophysical properties. This was clearly indicated by the bathochromic shift in the onset of absorption shown in hetero[8]circulene **49** compared to hetero[7]dehydrohelicenes **48**. In spite of their estimated high chiral stability (~38.5 kcal·mol^−1^), all attempts for their HPLC chiral resolution were unsuccessful preventing their further chiroptical study [75].

**Scheme 17:**
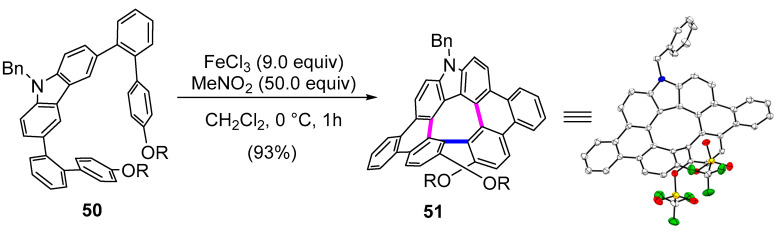
Facile synthesis of aza[7]dehydrohelicene **51** via intramolecular Scholl-type reaction.

In 2021, Maeda and Ema reported another dehydro[7]helicene **51** that can be prepared via an intramolecular FeCl_3_-mediated Scholl-type reaction from the precursor **50** (Scheme 17). The CPL of **51** was evaluated after HPLC chiral resolution, revealing |*g_lum_*| value of 2.5 × 10^−4^ at λ_em_ = 435 nm [76]. 

**Scheme 18:**
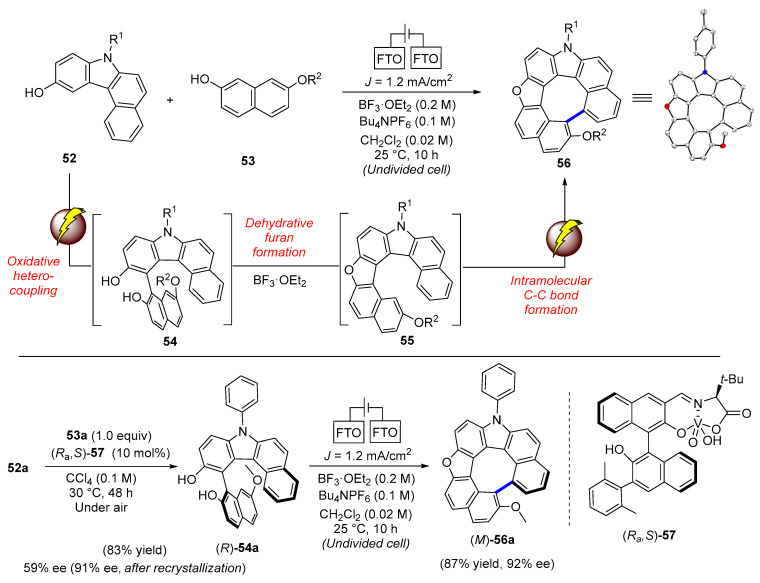
Sequential electrochemical synthesis of oxaza[7]dehydrohelicene: (**upper**) electrochemical one-pot process; (**lower**) stepwise enantioselective synthesis via chiral vanadium-catalyzed hetero-coupling and electrochemical oxidative transformations.

In 2022, Sasai and Takizawa introduced an efficient electrochemical sequential approach for the synthesis of diverse unsymmetrical oxaza[7]dehydrohelicenes **56**. Leveraging the readily available substrates: 3-hydroxy carbazoles **52** and 7-alkoxy-2-naphthol derivatives **53** as key coupling partners, their differential oxidation potentials (*E_ox_* of **52** = +0.69 V, and *E_ox_* of **53** = +1.32 V vs. Fc/Fc^+^ in CH_2_Cl_2_) played a crucial role in orchestrating the chemoselectivity of the oxidative heterocoupling step, resulting in diol derivatives **54**. Subsequent dehydrative cyclization of these diols yielded oxaza[7]helicene derivatives **55**, followed by an intramolecular C-C bond formation between the helical termini, ultimately furnishing the corresponding oxaza[7]dehydrohelicene derivatives **56** (Scheme 18) [10]. 

This protocol presents a highly efficient method for accessing diverse hetero[7]dehydrohelicenes **56** under mild conditions. The green electrochemical process sets itself apart by entirely eliminating metal waste, a notable departure from syntheses catalyzed by transitional metals [77,78,79,80,81,82]. The authors extended their approach to demonstrate the first enantioselective scalable synthesis of these dehydrohelicene molecules. This involves a stepwise hybrid chiral vanadium-catalyzed heterocoupling followed by electrochemical oxidative transformations (Scheme 18). To showcase the method’s applicability for concise synthesis, a two-pot protocol was tested using commercially available substrates *p*-benzoquinone and *N*-phenyl-2-naphthylamine to afford oxaza[7]dehydrohelicene derivatives **56** with an overall yield of up to 55% [10]. Later, the same group updated these electrochemical conditions to overcome some limitations and improve the Faradic efficiency side by side with the sustainability of their method [83]. 

The synthesized oxaza[7]dehydrohelicenes **56** exhibited notable properties, including intense blue-colored circularly polarized luminescence (|*g_lum_*| ≈ 2.5 × 10^–3^ at 433 nm), the highest CPL observed for all dehydrohelicenes so far, and significant chiral stability. Racemization barrier studies indicated ΔG^‡^ > 140 kJ·mol^−1^ and t_1/2_ > ca. 9.5 × 10^3^ years at 25 °C, discernibly higher level of chiral stability when juxtaposed with its oxaza[7]helicene counterparts (ΔG^‡^ > 110 kJ·mol^−1^ and t_1/2_ > ca. 5.7 days at 25 °C. The helical dyes within this study, demonstrated absorption within the wavelength range of 340–404 nm, coupled with fluorescence peaks at 420–450 nm [10].

**Scheme 19:**
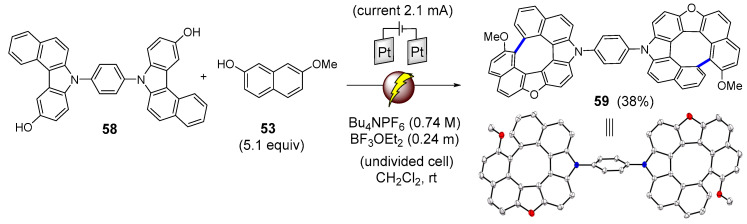
Data-driven electrochemical synthesis of double oxaza[7]dehydrohelicene.

In 2023, Salem and Takizawa employed this electrochemical approach to synthesize a novel heteronanographene with double chirality showcasing potential applications in various material-based technologies [84]. The study introduced an advanced Bayesian optimization (BO)-assisted screening of electrochemical conditions [85,86], efficiently identifying the optimum condition amidst the complexity of this sequential transformation with two functional sites and eight anodic events (Scheme 19). The unique architecture of this double oxaza[7]dehydrohelicene **59** exhibited high epimerization barriers (~34 kcal·mol^−1^), addressing a significant challenge in many multiple heteronanographenes—the low chiral stability.

The UV–VISs absorption of **59** was investigated using four solvents highlighting its excellent solubility in organic solvents, particularly advantageous for applications like solution-processed electronics [87,88]. The absorption patterns remained consistent across all solvents, with the highest absorbance at 323 nm and an optical bandgap (*E_g_*) of 2.98 eV observed in chloroform. Emission spectra were recorded in pure chloroform to reveal bathochromic shifts with emission maxima at λ**_em_** = 412 and 428 nm. The photoluminescence quantum yield (PLQY) of **59** measured at 30%, surpassing most reported chiral dehydrohelicenes. CD spectra indicate |*g_abs_*| values of 1.5 × 10^−3^, 2.5 × 10^−3^, and 2.1 × 10^−3^ at 398, 336, and 291 nm, respectively, for both enantiomers. Furthermore, |*g_lum_*| values for **59** measured as 1.5 × 10^−3^ at 430 nm, with (*P*,*P*)-**59** displaying a positive Cotton effect and (*M*,*M*)-**59** exhibiting a negative Cotton effect [84]. 

To this extent, it is evident that numerous synthetic approaches have been employed in the preparation of different dehydrohelicene-containing polycyclic compounds, as elucidated in Table 4. Most of these approaches relied on the Scholl reaction, either through utilizing an oxidizing agent such as (DDQ, chloranil, or FeCl_3_) side by side with the acid to prepare different compounds at lower temperatures, or through a traditional Scholl reaction employing Lewis acids (AlCl_3_) or protic acids (phosphoric acid) at elevated temperatures (>100 °C). Additionally, there was an example of employing the mechanochemical Scholl reaction for the production of compounds **17** and **18**. Some approaches relied on high temperatures (up to 290 °C), such as vacuum pyrolysis. Furthermore, metal-mediated oxidative coupling with metals such as tin, palladium, or molybdenum was introduced to furnish different compounds with dehydrohelicene core (**37**, **41**, and **42**). Other conditions for oxidative coupling using photochemical ring closure in the presence of iodine or hypervalent iodine (e.g., PIFA), have also been explored. Finally, a noteworthy example published by our groups employed electrochemical oxidation within a sequential framework to synthesize these compounds from readily available substrates.

Obviously, many of these dehydrohelicenes exhibited low racemization barriers, and in some cases (e.g., compounds **20** and **48**) researchers have faced challenges in achieving optical purity separation. This has hindered attempts to study their chiroptical properties. However, promising results have been demonstrated in studied examples such as compounds **51**, **56**, and **59** (Table 4).

## 4. Conclusions

This review encapsulates the synthesis and the photophysical and chiroptical characteristics of various molecules with dehydrohelicene cores. Initially considered as byproducts in the synthesis of circulenes half a century ago, dehydrohelicenes now exhibit significant potential in various material-based technologies. Many synthetic approaches to prepare dehydrohelicene-containing molecules have been discussed, yet many of them exhibit limitations such as low overall yields, harsh reaction conditions, or excessive use of oxidants. These challenges, coupled with difficulties in HPLC chiral resolution and low racemization barriers observed in certain instances, impede the progress of studies on the chiroptical behavior of these compounds. Despite recent advances introducing approaches that offer the potential for enantioselective production of these scaffolds, there remains a substantial need to investigate additional methodologies and derivatives, aiming to diversify the landscape of this chemistry. This need is particularly pronounced given the distinctive value conferred by these scaffolds, characterized by quasi-helical π-conjugated systems, non-planarity, exceptional optical properties, and intrinsic chirality. Recent advancements in material chemistry and the renewed interest in CPL-responsive small organic molecules have prompted a decade of scholarly efforts focused on developing facile synthesis methods for dehydrohelicenes. These compounds have a huge promise in applications spanning liquid crystals, material dyes, asymmetric synthesis, molecular switches, polymers, photorefractive materials, self-assembly processes, photovoltaics, light-emitting devices.

## Figures and Tables

**Table 1:** The photophysical properties of carbo[5]dehydrohelicene **2** in cyclohexane.

Compound	Ar	Abs (nm)	Em (nm)	Quantum Yield *ϕ*
**2a**	phenyl	436	478	0.51
**2b**	4-MeOC_6_H_4_	436	489	0.78
**2c**	4-ClC_6_H_4_	435	480	0.45
**2d**	4-CO_2_HC_6_H_4_	432	480	0.33
**2e**	4-NH_2_C_6_H_4_	436	478	0.58
**2f**	4-CHOC_6_H_4_	433	489	0.40
**2g**	4-CNC_6_H_4_	433	480	0.47
**2h**	3-thienyl	438	481	0.75

**Table 2:** The photophysical properties of carbo[5]dehydrohelicene **4** in THF.

Compound	Abs (nm)	Em (nm)	*ε*/M^−1^ cm^−1^	Stokes Shift
**4a**	311	481	48,300	170
**4b**	309	475	17,300	166
**4c**	308	474	43,100	166

**Table 3:** The photophysical properties of various thia[7]dehydrohelicenes in cyclohexane.

Compound	λ_max_/nm (ε/10^4^ mol^−1^ dm^3^ cm^−1^)			
**37**	244 (5.9)	326 (1.9)	338 (2.1)	348 (1.4)
**38**	265	-	-	-
**39**	256 (~3.3)	319 (~0.8)	333 (~0.8)	-

**Table 4:** Several approaches and chiroptical properties (*g_abs_*/*g_lum_*) of dehydrohelicenes.

Synthetic Approaches	Conditions	Compounds	Chiroptical Features *g_abs_*/*g_lum_*
Scholl reaction	DDQ-mediated Scholl reaction	**2**, **6**, **11**–**14, 23**–**25**	-
	Chloranil-mediated Scholl reaction	**48**	-
	FeCl_3_-mediated Scholl reaction	**51**	|*g_lum_*| value of 2.5 × 10^−4^ at λ_em_ = 435 nm
	Phosphoric acid at 110 °C	**4**	-
	AlCl_3_/NaCl at 140–150 °C	**28**, **30**–**35**	-
	Mechanochemical Scholl reaction	**17**, **18**	-
photochemical oxidative ring closure (*hv*, I_2_)		**20**	-
Vacuum pyrolysis	180–290 °C under a vacuum ≤ 0.1 mTorr	**37**	-
Metal-mediated oxidative coupling	*n*-Bu_3_SnH or Pd[P(*t*-Bu)_3_]_2_	**37**	-
	MoCl_2_	**41**, **42**	Cotton effects at 501, 471, 421 nm
Oxidative fusion reaction	DDQ-Sc(OTf)_3_	**44a**, **44b**	-
Hypervalent Iodine-induced Cyclization	PIFA/NaBH_4_	**44c**	Cotton effects at 450, and 340 nm
Electrochemical oxidation		**56**, **59**	|*g_lum_*| of **56** = 2.5 × 10^−3^ |*g_lum_*| of **59** = 1.5 × 10^−3^

## Data Availability

Not applicable.
